# Multi-scale fusion visual attention network for facial micro-expression recognition

**DOI:** 10.3389/fnins.2023.1216181

**Published:** 2023-07-27

**Authors:** Hang Pan, Hongling Yang, Lun Xie, Zhiliang Wang

**Affiliations:** ^1^Department of Computer Science, Changzhi University, Changzhi, China; ^2^School of Computer and Communication Engineering, University of Science and Technology Beijing, Beijing, China

**Keywords:** micro-expression recognition, attention mechanism, mask operate, multi-scale feature, feature fusion

## Abstract

**Introduction:**

Micro-expressions are facial muscle movements that hide genuine emotions. In response to the challenge of micro-expression low-intensity, recent studies have attempted to locate localized areas of facial muscle movement. However, this ignores the feature redundancy caused by the inaccurate locating of the regions of interest.

**Methods:**

This paper proposes a novel multi-scale fusion visual attention network (MFVAN), which learns multi-scale local attention weights to mask regions of redundancy features. Specifically, this model extracts the multi-scale features of the apex frame in the micro-expression video clips by convolutional neural networks. The attention mechanism focuses on the weights of local region features in the multi-scale feature maps. Then, we mask operate redundancy regions in multi-scale features and fuse local features with high attention weights for micro-expression recognition. The self-supervision and transfer learning reduce the influence of individual identity attributes and increase the robustness of multi-scale feature maps. Finally, the multi-scale classification loss, mask loss, and removing individual identity attributes loss joint to optimize the model.

**Results:**

The proposed MFVAN method is evaluated on SMIC, CASME II, SAMM, and 3DB-Combined datasets that achieve state-of-the-art performance. The experimental results show that focusing on local at the multi-scale contributes to micro-expression recognition.

**Discussion:**

This paper proposed MFVAN model is the first to combine image generation with visual attention mechanisms to solve the combination challenge problem of individual identity attribute interference and low-intensity facial muscle movements. Meanwhile, the MFVAN model reveal the impact of individual attributes on the localization of local ROIs. The experimental results show that a multi-scale fusion visual attention network contributes to micro-expression recognition.

## 1. Introduction

Human-computer interaction not only requires machines to complete specified tasks, but also requires machines to have emotional cognition, communication, and feedback capabilities like humans during the interaction process (Ahmad et al., 2019). Human emotions can be expressed through speech (Zhao et al., 2021), text (Wang et al., 2020b), gestures (Li Y. -K. et al., 2022), and physiological signals (Wu et al., 2023), but facial expressions most intuitively reflect human emotions. The research has found that people would intentionally display certain facial expressions in certain situations, yet when people try to hide their facial expressions in high-stakes situations, it is necessary to interpret facial micro-expressions to determine their true emotional state (Ekman and Friesen, 1969).

Facial micro-expressions (hereinafter referred to as micro-expressions) are short-duration and low-intensity facial muscle movements. Since it is usually caused by suppressed emotions and can result from genuine motivations and emotions (Ekman, 2009). If people are not professionally trained, it is impossible to hide the appearance of micro-expressions (Holler and Levinson, 2019). Researchers found that micro-expressions are often present in lie detection scenarios. Thus, it has major implications when it comes to high-risk situations including criminal investigation, social interactions, national security, and business negotiations (O’Sullivan et al., 2009). It is less accurate to recognize micro-expressions (Ben et al., 2021; Tran et al., 2021; Zhou et al., 2021; Bisogni et al., 2022; Wang et al., 2022; Zhu et al., 2022; Wei et al., 2022a) than facial expressions (Shao and Qian, 2019; Li and Deng, 2020; Chowdary et al., 2021; Bisogni et al., 2022). The current research on micro-expression recognition is still correlating datasets collected the laboratory environments, which is not enough for application in high-risk scenes.

**Figure 1:**
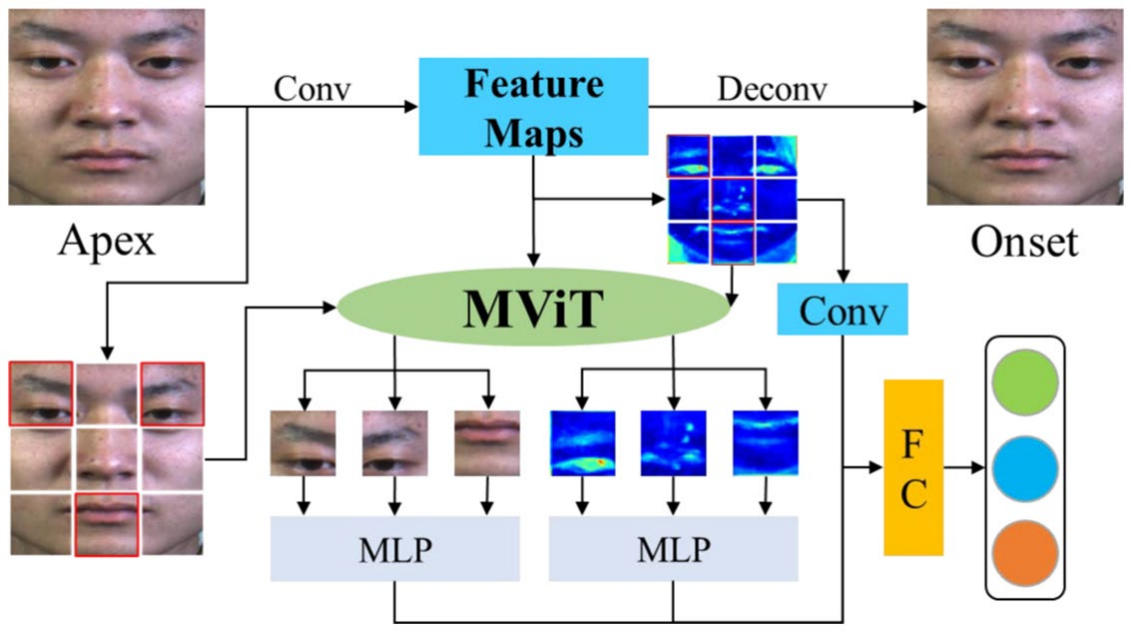
The framework of MFVAN model to explores the effect of reducing individual identity attributes on emotional change ROIs localization and learns multi-scale local attention weights to mask regions of redundancy features.

With the rapid development of image acquisition equipment, researchers use high-speed cameras to collect micro-expression images to create the Spontaneous Micro-Expression Database (SMIC) (Li et al., 2013), Chinese Academy of Sciences Micro-expression database (CASME II) (Yan et al., 2014), Spontaneous Actions, and Micro-Movements (SAMM) (Davison et al., 2016), Micro-and-Macro Expression Warehouse (MMEW) (Ben et al., 2021), and Third Generation Facial Spontaneous Micro-Expression Database (CAS (ME)^3^) (Li J. et al., 2022). These datasets are collected by high-speed cameras to alleviate the problem of short duration. However, facial muscle movements low-intensity are still an important factor inhibiting the enhancement accuracy of micro-expression recognition. For the low-intensity challenge of micro-expressions, researchers extract efficient local features for micro-expression recognition through regions-of-interest (ROIs) localization prior knowledge (Xu et al., 2017; Niu et al., 2019; Yu et al., 2019; Merghani and Yap, 2020) or local ROIs localization based on deep learning (DL) (Bai, 2020; Chen et al., 2020; Xia et al., 2020; Xie et al., 2020; Wang et al., 2020a; Li et al., 2021; Zhao et al., 2022). Although the local features extraction after local ROIs locating through prior knowledge or deep learning methods is helpful for micro-expression recognition, but these methods ignore the feature redundancy caused by the inaccuracy of ROIs. Moreover, psychological research has shown that muscle movement changes during facial expression did not correlate with individual identity attributes such as gender, age, and ethnicity (Ekman and Friesen, 1971). However, these micro-expression recognition methods do not consider the effect of individual identity attributes on the localization of ROIs. To overcome the challenge of low-intensity facial muscle movements in the micro-expression recognition task, this paper proposes a novel multi-scale fusion visual attention network (MFVAN). This model explores the effect of reducing individual identity attributes on emotional change ROIs localization and learns multi-scale local attention weights to mask regions of redundancy features. The framework of the MFVAN model is shown in Figure 1.

The micro-expression image (apex frame) is input into the convolutional neural network (CNN) that is mapped to a calm state image (onset frame) to reduce the effect of individual identity attributes and obtain multi-scale feature maps. The multi-head self-attention (MSA) extracts the local features of the multi-scale feature maps and obtains the attention weights of these features. We reduce feature redundancy by dropping out local features irrelevant to micro-expressions according to attention weights. At the same time, local features with higher attention weights in multi-scale are fused to improve the robustness features. Finally, the multi-scale classification loss, mask loss, and removing individual identity attributes loss joint to optimize the MFVAN model. The experimental results on SMIC, CASME II, SAMM, and their combined datasets (See et al., 2019) demonstrate that the MFVAN can achieve state-of-the-art performance by fusing multi-scale local attention features. Overall, our proposed MFVAN model is the first to combine image generation with visual attention mechanisms to solve the combination challenge problem of individual identity attribute interference and low-intensity facial muscle movements. In summary, the main contributions of this paper can be summarized as follows:

1. This paper analyzed the combined effects of facial identity attributes on micro-expression recognition. This paper analyzes the combined effects of low-intensity of facial muscle movement changes and individual identity attributes in micro-expression recognition.
2. This paper is the first study that combines image generation with visual attention mechanisms and proposes an MFVAN framework. The self-supervised and transfer learning is jointly trained to remove individual identity attributes.
3. Meanwhile, the MFVAN model utilizes the global and multi-scale local attention weights connected for micro-expression recognition. The focal loss, removing identity attributes loss, and the marked loss are used to optimize the MFVAN model. The experimental results on SMIC, CASME II, SAMM, and their combined datasets demonstrate that the MFVAN can achieve state-of-the-art performance that focuses on local at the multi-scale and contributes to micro-expression recognition.

The rest of this paper is structured as follows: In Section 2, we review the micro-expression recognition datasets, handcrafted features, and deep learning micro-expression recognition methods. Section 3 presents the proposed MFVAN framework. Section 4 presents the experimental dataset, evaluation metrics, quantitative analysis, and analysis of ablation experiments. Finally, Section 5 summarizes the proposed algorithm and discusses future research trends.

## 2. Related research

This section introduces the facial micro-expression recognition dataset used in the experiments part. Then, by comparing the research status of micro-expression recognition based on handcrafted features and deep learning, the shortcomings of existing research are obtained that laying the groundwork for the research method.

### 2.1. Datasets description

The premise of micro-expression recognition must have sufficient data with emotional labels. However, the research on facial micro-expression recognition through computer vision has just started. At present, there are very few micro-expression datasets, mainly including imitation and spontaneous datasets. The most important difference between the two is the correlation of facial micro-expression manifestations with underlying emotional states. Among them, spontaneous micro-expressions are facial movements shown through external stimuli, which are consistent with underlying emotional states. Therefore, this paper adopts three spontaneous facial micro-expression datasets and their combined datasets to verify the proposed MFVAN method.

The SMIC is the first public dataset used for micro-expression recognition. This dataset includes 328 videos collected from 20 subjects. During the experiment, a high-speed camera with a resolution of 640 × 480 pixels and a transmission rate of 100 frames per second (FPS) was used to collect the facial images of each subject throughout the process. The researchers screened 164 video clips (negative, positive, surprise) inspired by all 16 subjects participating in the experiment to form SMIC for facial micro-expression recognition. The CASME II was recorded with a camera with 640 × 480 pixels and 200 FPS from 26 subjects. The researchers screened 246 video clips (happiness, surprise, disgust, repression, others) that were selected from more than 3000 facial actions. The SAMM is a high-speed and high-resolution dataset that uses a camera with a frame rate of 200 and a resolution of 2040 × 1088 to capture images. The database collected video data from 32 subjects with an average age of 33.24 years by spontaneous elicitation, with an even and rich ethnic distribution. The 159 samples were labeled including happiness, surprise, contempt, anger, others, disgust, fear, and sadness. The 3DB-combined dataset is a reclassification and combination of CASME II and SAMM based on SMIC dataset labels. Among them, depression, sadness, contempt, and disgust are divided into negative categories in the SMIC dataset, and happiness is divided into positive categories. The recombined 3DB-combined dataset contains 442 samples among which 109 positive, 250 negative, and 83 surprised, including all 164 samples in SMIC, 145 samples in CASME II, and 133 samples in SAMM.

### 2.2. Handcrafted features methods

Facial micro-expression recognition methods are generally divided into two categories, one is to extract the manual change features of facial images of micro-expression video sequences for micro-expression recognition, and the other is to first use deep learning methods for micro-expression recognition. In the previous facial micro-expression recognition, to describe the changes of micro-expressions, many research works have used manual feature-based methods to extract the changes in texture, color, and optical flow characteristics of image sequences, splicing them into a compact feature vector and outputting it to the classifier identifies micro-expressions.

The Local Binary Pattern from Three Orthogonal Planes (LBP-TOP) (Pfister et al., 2011) is the representative handcrafted feature extraction method applied to micro-expression recognition. The follow-up research work is the improvement of LBP-TOP. The Local Binary Pattern Six Interception Points (LBP-SIP) (Wang et al., 2014) and Local Binary Pattern from Mean Orthogonal Planes (LBP-MOP) (Wang et al., 2015) are used to reduce the redundancy problem. The Kernelized Two-Groups Sparse Learning (KTGSL) (Wei et al., 2022b) automatically learns more discriminative features from Local Binary Pattern with Single Direction Gradient (LBP-SDG) (Wei et al., 2021) and Local Binary Pattern from Five Intersecting Planes (LBP-FIP) (Wei et al., 2022a) two sets of features to improve micro-expression recognition performance. The Discriminative Spatiotemporal Local Radon Binary Pattern Based on Revisited Integral Projection (DiSTLBP-RIP) (Huang et al., 2019) fuses shape features into LBP-TOP to improve the ability to discriminate micro-expressions.

In addition to texture and shape features, optical flow features are also manual features commonly used in micro-expression recognition (Li et al., 2020). The Fuzzy Histogram of Optical Flow Orientation (FHOFO) (Happy and Routray, 2017) is employed the Facial Action Coding System (FACS) to locate 36 facial ROIs to extract the subtle changes in these regions for micro-expression recognition. The Weighted Oriented Optical Flow (BI-WOOF) (Liong et al., 2018) weighted average of the overall and local histogram of oriented optical flow features. The Sparse Main Directional Mean Optical Flow (SMDMO) (Liu et al., 2018) averages the optical flow features of the region of interest of 36 motion units in the face area to reduce the noise effect caused by head movement in micro-expression recognition. Although the method based on handcrafted features can achieve good performance in micro-expression recognition, it requires a lot of preprocessing such as face detection alignment and video frame insertion in the early stage. With the application of end-to-end deep learning methods in the field of image recognition, more and more researchers have begun to consider how to use deep learning methods to solve micro-expression recognition tasks.

### 2.3. Deep learning methods

With the development of DL, especially the proposal of the CNN model which includes AlexNet (Krizhevsky et al., 2012), GoogLeNet (Szegedy et al., 2015), ResNet (He et al., 2016), and SENet (Hu et al., 2018) for image recognition. With the application of DL in face detection, face recognition, face editing, and expression recognition, more researchers have begun to pay attention to micro-expression recognition through DL methods. Kim et al. (2016) is the first attempt to use the combination of CNN and Long Short-Term Memory (LSTM) to extract spatiotemporal features of micro-expression video sequences for MER.

Gan et al. feed apex optical flow (OFF-Apex) (Gan et al., 2019) images into CNN for micro-expression recognition. The Dual-Stream Shallow Network (DSSN) (Khor et al., 2019)model reduces the complexity of the model by pruning the CNN model while improving the recognition performance of micro-expressions. The Stacked Hybrid Convolution Feature Network (SHCFNet) (Huang et al., 2020) enhances the CNN network with optical flow features of different scales. The GEender-based Micro-Expression (GEME) (Nie et al., 2021) reduces the impact of gender on CNN models through a two-stream multi-task framework. The Local and Global information joint learning module (LGCcon) (Li et al., 2021) localizes the main emotional information local area while suppressing the negative impact of irrelevant local areas on micro-expression recognition. The Action Unit – Graph Convolution Network (AU-GCN) (Lei et al., 2021) enhances the feature representation of nodes and graph edges extracted by the graph convolutional network by fusing AU coding features. The Two-Stream Graph Attention Convolutional Network (TSGACN) (Kumar and Bhanu, 2021) encodes features and optical flow features by fusing facial key points. The (Feature Refinement, FeatRef) (Zhou et al., 2022) framework uses the attention model to select the obvious discriminant features for micro-expression recognition. The Prototypical Learning with Local Attention Network (PLAN) (Zhao et al., 2022) learns local facial action change features through local attention modules.

These deep learning techniques can improve recognition performance by learning more efficient depth features than hand-crafted features from video sequences or the apex frame of micro-expression samples. However, these works rarely consider the impact of local information. Although the AU-GCN, TSGACN, and PLAN methods use AU information to assist micro-expression recognition, they do not consider the influence of individual identity attributes on local information.

## 3. Methods

**Figure 2:**
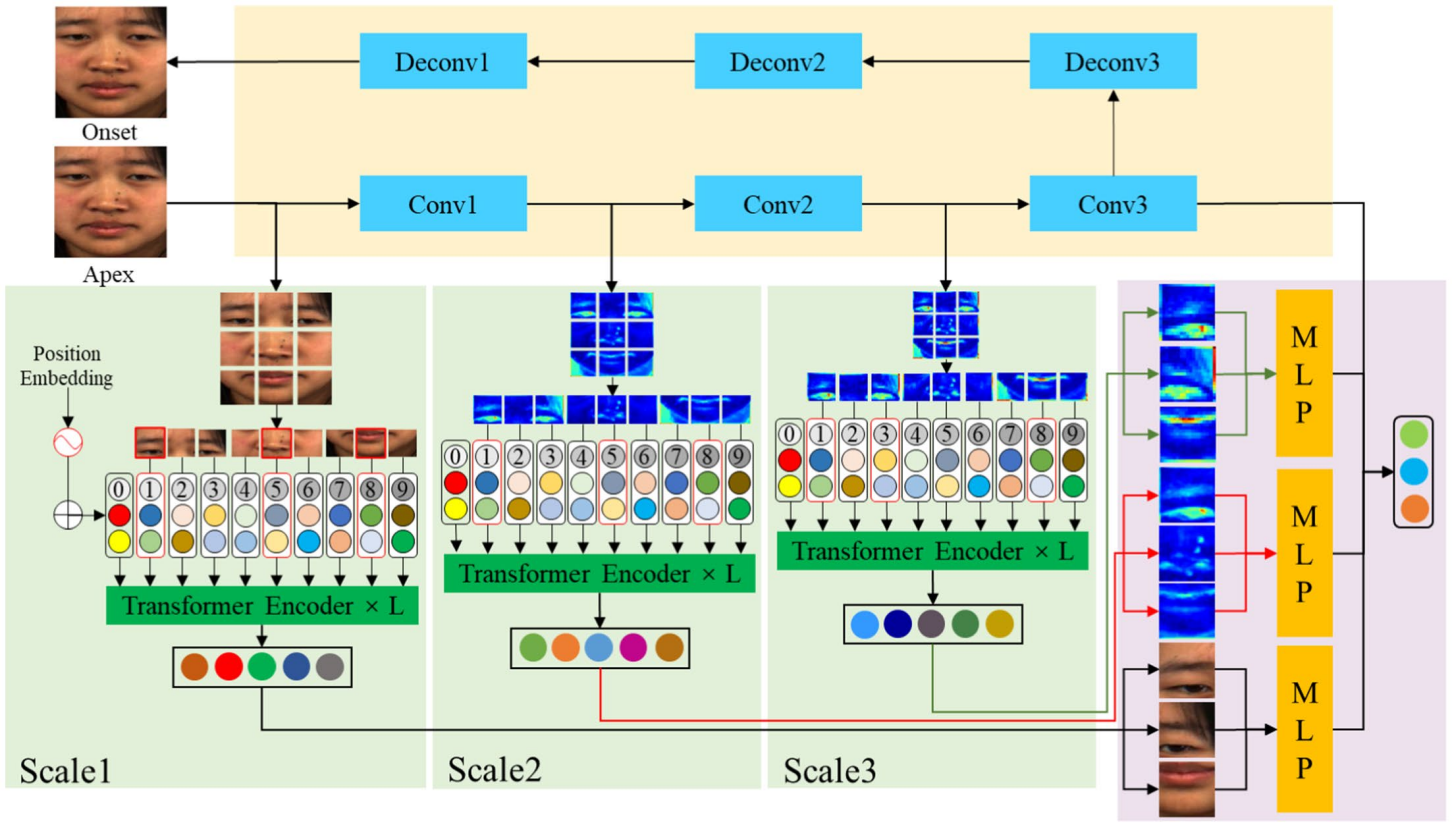
The MFVAN model contains three components. The first is a de-identity attribute model based on Convolutional Neural Network (CNN) to extract global facial features. Then multi-scale vision transformer (MViT) model is used to capture local information about the apex frame and different convolutional layers where the red boxes represent regions with higher weights. Finally, multi-scale local features with high attention weight are fused for micro-expression recognition.

In this paper, our proposed network extracts global features by removing individual identity attributes. Then multi-scale attention mechanism is used to capture local information of the global image and different convolutional layers. Finally, the multi-scale local features with high weights are fused to classify the apex frame. The architecture of MFVAN is illustrated in Figure 2, our proposed method framework MFVAN is a multi-scale joint network.

### 3.1. Removing identity attributes

Psychologists believe that facial micro-expressions are not related to individual identity attributes. Meanwhile, Valstar and Pantic (2011) consider that the onset frame in the micro-expression video sequence represents the moment when the appearance of the face is enhanced. So, the onset frame can be considered as an identity image, which contains the identity attributes of the individual. The apex frame is the coupling of individual identity attributes and emotional representation. In the process of decoupling identity information, we map the apex frame to the onset frame through the autoencoder model. The encoder module is used to map apex frames to identity features. Then the identity features are used to generate an identity image through the decoder module. Therefore, we map the apex frame (facial micro-expression images) into CNN to the onset frame (neutral states image) to remove the identity information and obtain the global change features.

**Figure 3:**
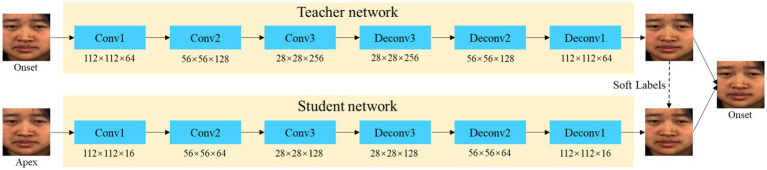
Illustration of the transfer learning framework for removing identity attributes.

The problem of a small dataset of micro-expressions severely constrains the training of the mapping model for removing identity attributes. To better learn the CNN mapping model, we use self-supervised and transfer learning to train the mapping model. We train the teacher model by a deep image self-supervision approach. The image is mapped to a high-dimensional feature space using multiple residual modules at the image encoder and then returned to the original image by deconvolution. The teacher networks are complex with superior performance. Then a shallow network student network is designed to learn the mapping relationship of the apex to the onset frame. This teacher network is used as a soft target to guide shallow student networks so that a simpler student model with fewer parameters can have a similar performance as the teacher network. The network structure is shown in Figure 3.

In the training process, we first pre-trained the teacher model. Image self-supervised training is performed by feeding all images from the micro-expression video samples into the teacher model. Then the teacher and student network are jointly trained. The apex frame input generates a network to output the onset frame. The model parameters are optimized by the mean squared error (MSE) loss function of the two models. The loss functions for the pre-training model and the removing identity attribute model are $L_{pre}$ and $L_{remove}$ , which are computed as follows:


(1) $$L_{pre}=\frac{1}{N*M}\overset{N}{\underset{i=1}{\sum }}\overset{M}{\underset{j=1}{\sum }}{\left|x_{i,j}-x_{i,j}^{\prime }\right|}^{2}$$

(2) $$L_{Teacher}=\frac{1}{N*M}\overset{N}{\underset{i=1}{\sum }}\overset{M}{\underset{j=1}{\sum }}{\left|x_{onset}{}_{i,j}-x_{onset}^{\prime }{}_{i,j}\right|}^{2}$$

(3) $$L_{student}=\frac{1}{N*M}\overset{N}{\underset{i=1}{\sum }}\overset{M}{\underset{j=1}{\sum }}{\left|x_{onset}{}_{i,j}-x_{onset}^{\prime \prime }{}_{i,j}\right|}^{2}$$

(4) $$L_{remove}=L_{Teacher}+L_{student}$$

where x is the image of micro-expression video samples. $x^{\prime }$ is the image generated by the pre-trained model. $x_{onset}$ is the onset frame. $x_{onset}^{\prime }$ and $x_{onset}^{\prime \prime }$ is the image generated by the teacher and student network.

### 3.2. Multi-scale fusion visual attention network

**Figure 4:**
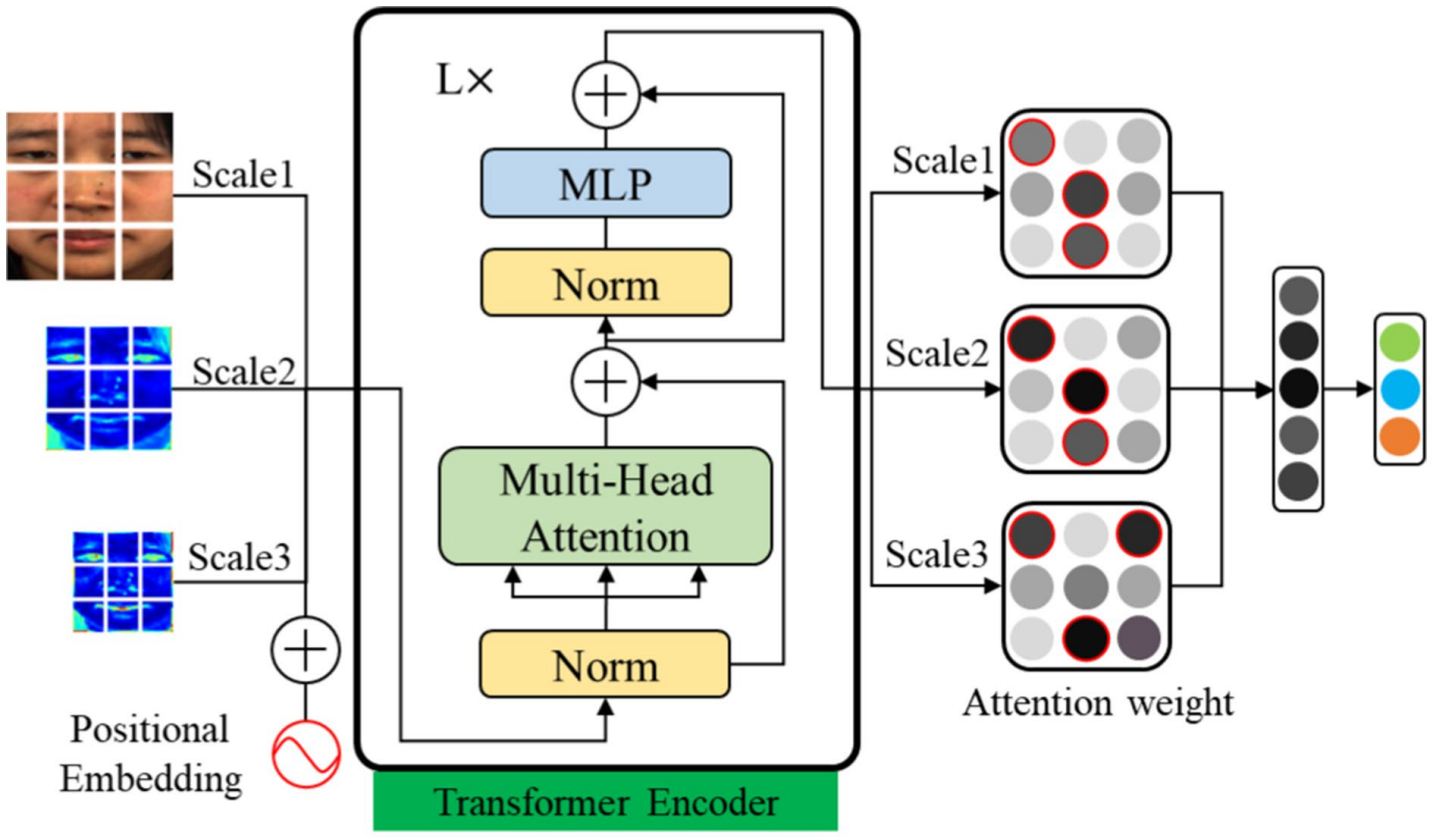
The architecture of MViT model. The apex frame and multi-scale feature are input transformer encoders. Then the local feature with high weights on each scale is fused.

The low-intensity characteristic of micro-expressions represent as muscle movement changes in localized regions of facial images. However, inaccurate localization of local regions can lead to feature redundancy and thus affect recognition performance. In this paper, we propose an MFVAN model for improving micro-expression recognition performance by extracting the local features of the multi-scale feature map. The feature weights of the apex frame and the patch token of the feature map at multiple scales are learned by MSA in the visual transformer model (Dosovitskiy et al., 2021). Then the patch token with a high weight at each scale is input into multi-layer perceptron (MLP) fusion to recognize micro-expressions. The MFVAN structure is shown in Figure 4.

The MFVAN model flattens the apex frame and multi-scale feature maps are split into s×s patches and flattened to generate image sequences $x_{i}$ . These image patches sequence is mapping to a feature vector $f_{i}$ with convolution operating and weighting the positional embedding $f_{pos}$ to generate a new feature vector. The dimension of $f_{i}$ is k×q . The parameter k is s×s which is the image patches token length. The dimension q is determined by the convolution mapping performed on each block to generate the feature dimension. For each scale feature vector, we add a class token. The calculation process of the new feature vector $f_{i}^{\prime }$ of the i−th scale is shown in Eq. (5).


(5) $$f_{i}^{\prime }=\left[f_{c,i},f_{i}+f_{pos}\right],i\in \left(1,2,3\right)$$

(6) $$f_{i}=F_{conv}\left(\sum x_{i}w_{i}+b_{i}\right)$$

where $f_{c,i}$ is the class token of the i−th scale, $f_{i}$ is the feature by convolution mapped, $f_{pos}$ is the positional embedding, i is the scale, $w_{i}$ and $b_{i}$ are the weights of the convolution mapped of the i−th scale.

In the transformer encoder model, the class token is a learnable classification parameter. But in the MFVAN model, the class token is not only used for classification, but also used to learn the attention weight of each patch, and perform mask operation on the patch with low weight. The transformer encoder module in the MFVAN model contains L layers of MSA and MLP blocks. The feature vectors corresponding to patches with attention weights greater than θ are fused by the MLP module for classification. The classification process is shown in Eq. (7).


(7) $$p=SoftMax\left(F_{MLP-f}\left(\frac{1}{3}\overset{3}{\underset{i=1}{\sum }}f_{i,L}^{\prime }\right)\right),f_{c}\geq \theta$$

(8) $$f_{i,l}^{\prime }=F_{MLP,l}\left(F_{MSA,l}\left(f_{i,l-1}^{\prime }\right)\right),l\in 1,2,\ldots ,L$$

where p the prediction result of the MFVAN model, $F_{MLP-f}$ is the fused function, $\;f_{i,0}^{\prime }$ is the input embedding vector, $f_{i,L}^{\prime }$ is the output of the transformer encoder module of the i−th scale, $f_{c}$ is the attention weight, θ is the threshold for dividing the attention weights, L is the number of MSA and MLP blocks, $F_{MSA,l}$ and $F_{MLP,l}$ are the *l-th* layer block.

### 3.3. Loss function optimization based on global and local

In this paper, we use removing identity attributes loss, global classification loss, multi-scales classification loss, and multi-scales mask loss function joint to optimize the MFVAN model and learn the local patch attention weight when the micro-expression occurs.


(9) $$L_{all}=L_{remove}+L_{class\_global}\left(p,y\right)+\overset{3}{\underset{i=1}{\sum }}L_{class\_scale,i}\left(p_{i,scale,},y\right)+L_{mask}$$

(10) $$L_{class}=-\alpha_{t}{\left(1-p_{t}\right)}^{r}\log\left(p_{t}\right)$$

(11) $$L_{mask}=\{\begin{array}{cc} 0, & if\;f_{c}<\theta \\ f_{c}, & otherwise \end{array}$$

where $L_{remove}$ is the removing identity attributes loss, $L_{class\_global}$ is the global level of classification loss, $L_{class\_scale,i}$ is the classification loss of the i−th scale, p is the probability of the global prediction, $p_{i,scale,}$ is the probability of i−th scale, y is the ground truth, $L_{class}$ is the classification loss, $L_{mask}$ is mask loss, $\alpha_{t}$ , r are hyperparameters. $\alpha_{t}$ represents the weight of the t-th class sample, and $p_{t}$ represents the probability value of the *t*-th class output by Softmax.

## 4. Experimental analysis

In this section, the evaluation metrics, comparative analysis of experimental results, ablation experiments, and visualization analysis will be introduced in detail. The proposed MFVAN method is evaluated on SMIC, CASME II, SAMM, and 3DB-Combined datasets.

### 4.1. Evaluation metric

The evaluation metric for micro-expression recognition is the accuracy and F1-score on the single dataset by the Leave-One-Subject-Out (LOSO) cross-validation. The Unweighted F1-score (UF1) and Unweighted Average Recall (UAR) on the combined datasets. The evaluation metric is computed using:


(12) $$F1^{\_}score=2\times \frac{Precision\times Recall}{Precision+Recall}$$

(13) $$Recall=\frac{TP}{TP+FN}$$

(14) $$Precision=\frac{TP}{TP+FP}$$

(15) $$Accuracy=\frac{TP+TN}{TP+FP+TN+FN}$$

(16) $$UF1=\frac{1}{C}\overset{C}{\underset{c=1}{\sum }}F1_{c}$$

(17) $$UAR=\frac{1}{C}\sum Acc_{c}$$

where TP is true positive, TN is true negative, FP is the false positive, and FN is false negative, C is the total number of categories.

### 4.2. Result analysis of a single datasets

In this part, we evaluate the effectiveness of the MFVAN model by comparing it with two types of baseline methods based on handcrafted features and deep learning on the single dataset. In the comparison experiment of handcrafted feature methods, this paper selects the most representative LBP-TOP, BI-WOOF, DiSTLBP-RIP, LBP-SDG, and KTGSL with our proposed MFVAN model contrasted. In the deep learning method comparison experiment, we chose the OFF-Apex, DSSN, LGCcon, GEME, AU-GCN, and FeatRef models. The Bi-WOOF method based on handcrafted features and the AU-GCN model based on deep learning adopt the method of ROIs positioning. The experimental results are shown in Table 1.

**Table 1:** Micro-expression recognition performance comparison on the SMIC (3 categories), CASME II (5 categories), and SAMM (5 categories).

Methods	SMIC (3)		CASME II (5)		SAMM (5)	
	Accuracy	F1-Score	Accuracy	F1-Score	Accuracy	F1-Score
LBP-TOP (2011)	48.78	0.4600	39.68	0.3589	35.56	0.3589
Bi-WOOF (2018)	61.59	0.6110	57.89	0.6125	–	–
DiSTLBP-RIP (2019)	63.41	–	64.78	–	–	–
LBP-SDG (2021)	69.68	0.6200	71.32	0.6700	–	–
KTGSL (2022)	**75.64**	**0.6900**	**72.58**	**0.6800**	**56.11**	**0.4900**
OFF-Apex (2019)	**67.68**	**0.6709**	68.94	0.6967	–	–
DSSN (2019)	63.41	0.6462	70.78	0.7297	57.35	0.4644
LGCcon (2021)	–	–	65.02	0.6400	40.90	0.3400
GEME (2021)	64.63	0.6158	**75.20**	**0.7354**	55.88	0.4538
AU-GCN (2021)	–	–	74.27	0.7047	**74.26**	**0.7045**
FeatRef (2022)	57.90	–	62.85	–	60.13	–
MFVAN	**79.87**	**0.8009**	**78.45**	**0.7616**	**76.47**	**0.7325**

The bold values represent the state-of-the-art performance of Handcrafted features and deep learning methods, as well as the performance of our proposed MFVAN method.

The experimental comparison in the SMIC dataset found that the accuracy of MFVAN was 4.23 and 12.19% higher than the best KTGSL in handcrafted features and the best OFF-Apex in deep learning. The performance of the F1-Score is 0.1109 and 0.13 higher, respectively. The MFVAN model also achieves state-of-the-art performance on two other single CASME II and SAMM datasets. In all comparative experimental analyses, almost all methods input video sequences of micro-expression samples, only OFF-Apex, DSSN, GEME, and AU-GCN methods use apex frame (or apex frame and onset frame) for micro-expression recognition. The OFF-Apex model is one of the representative methods that only use peak frame information for deep learning training in the early stage of micro-expression recognition. Most of the subsequent methods are based on it to improve and improve the model or method. For example, DSSN compresses the model by pruning, and GEME eliminates the influence of individual gender. Although GEME has considered the interference of gender, their limitation is that it only considers the interference of gender, and the individual identity attribute has the influence of other attributes such as skin color and age in addition to gender. Therefore, the MFVAN model self-supervision and transfer learning reduce the influence of individual identity attributes and increase the robustness of multi-scale feature maps to improve the performance of micro-expression recognition.

### 4.3. Result analysis of a combined dataset

This section also further verifies the effectiveness of the MFVAN model on the 3DB-Combined dataset of the MEGC 2019. Since DiSTLBP-RIP, LBP-SDG, KTGSL, and DSSN do not report experimental results on combined datasets, we conduct comparative experiments with the remaining methods. It is worth noting that since the SMIC dataset does not provide the marker of the peak frame, in the comparison experiment of the composite dataset, the LGCcon model only reports the experimental results of the adjusted dataset. The experimental results are shown in Table 2.

**Table 2:** Micro-expression recognition performance comparison on the 3DB-combined datasets.

Methods	SMIC		CASME II		SAMM		3DB-combined	
	UF1	UAR	UF1	UF1	UF1	UF1	UF1	UAR
LBP-TOP (2011)	0.2000	0.5280	0.7026	0.5882	0.5882	0.7026	0.3954	0.4102
Bi-WOOF (2018)	0.5727	0.5829	0.7805	0.6296	0.6296	0.7805	0.5211	0.5139
OFF-Apex (2019)	0.6817	0.6695	0.8764	0.7196	0.7196	0.8764	0.5409	0.5409
GEME (2021)	0.6288	0.6570	0.8401	0.7395	0.7395	0.8401	0.6868	0.6541
LGCcon (2021)	0.6195	0.6066	0.7762	0.7499	0.4924	0.4711	–	–
AU-GCN (2021)	**0.7192**	**0.7215**	0.8798	**0.7914**	**0.7914**	0.8798	**0.7751**	**0.7890**
FeatRef (2022)	0.7011	0.7083	**0.8915**	0.7838	0.7838	**0.8915**	0.7372	0.7155
MFVAN	**0.7986**	**0.7899**	**0.9061**	**0.8100**	**0.8100**	**0.9061**	**0.8322**	**0.8289**

The bold values represent the state-of-the-art performance of the deep learning method and our proposed MFVAN method.

The first 6 columns are the experimental results of the adjusted three-category dataset. Similar to the original dataset, the MFVAN model can achieve competitive results, and the UF1 and UAR indicators are 0.0794/0.0684, 0.0263/0.0186, and 0.0186/0.0263 higher than the optimal AU-GCN model on the two datasets. The MFVAN can achieve state-of-the-art performance in the combined dataset.

### 4.4. Ablation experiment analysis

To evaluate the effectiveness of the MFVAN model, we conducted ablation experiments analysis comparison of attention models at different scales on SMIC, CASME II, and SAMM. The detailed experimental comparisons of global features (Global), global features with removed identity attributes (Global+RI), and fusion with local features at different scales (Global+scale). The results are shown in Table 3. The experiment found that removing the interference of identity information by the apex to the onset frame mapping method can improve the performance of Accuracy and F1-Score, both in global features and global features fused with multi-scale local features. This situation also illustrates that removing the identity attributes can optimize the recognition performance of micro-expressions on SMIC, CASME II, and SAMM.

**Table 3:** Evaluation for global and local features on the SMIC (3 categories), CASME II (5 categories), and SAMM (5 categories).

Methods	SMIC (3)		CASME II (5)		SAMM (5)	
	Accuracy	F1-Score	Accuracy	F1-Score	Accuracy	F1-Score
Global	59.88	0.5964	62.29	0.5860	40.35	0.4056
Global+RI	62.67	0.6274	63.23	0.6288	43.01	0.4240
Global+scale1	64.03	0.6394	76.55	0.7614	71.72	0.7061
Global+scale1 + RI	66.41	0.6661	**79.45**	**0.7816**	74.32	0.7164
Global+scale1,2	67.47	0.6851	75.39	0.7427	75.11	0.7137
Global+scale1,2 + RI	72.15	0.7155	75.71	0.7500	75.89	0.7267
Global+scale1,2,3	70.10	0.6941	75.92	0.7482	75.32	0.7297
Global+scale1,2,3 + RI	**79.87**	**0.8009**	78.45	0.7616	**76.47**	**0.7325**

The bold values represent the optimal performance compared to ablation experiments.

At the same time, we found that the performance of micro-expression recognition increases accordingly with the fusion of local features at multiple scales. In the SMIC and SAMM, multi-scale local feature fusion can better capture the local detail changes of the apex to the onset frame and improve the recognition performance of micro-expressions. However, we achieve the best performance by fusing Accuracy and F1-Score with the original scales in CASME II, which are 79.45 and 0.7816, respectively. Therefore, for the consistency of experimental results across all datasets, we use multi-scale fusion to obtain the final experimental results in the experimental validation process.

### 4.5. Visualization analysis

**Figure 5:**
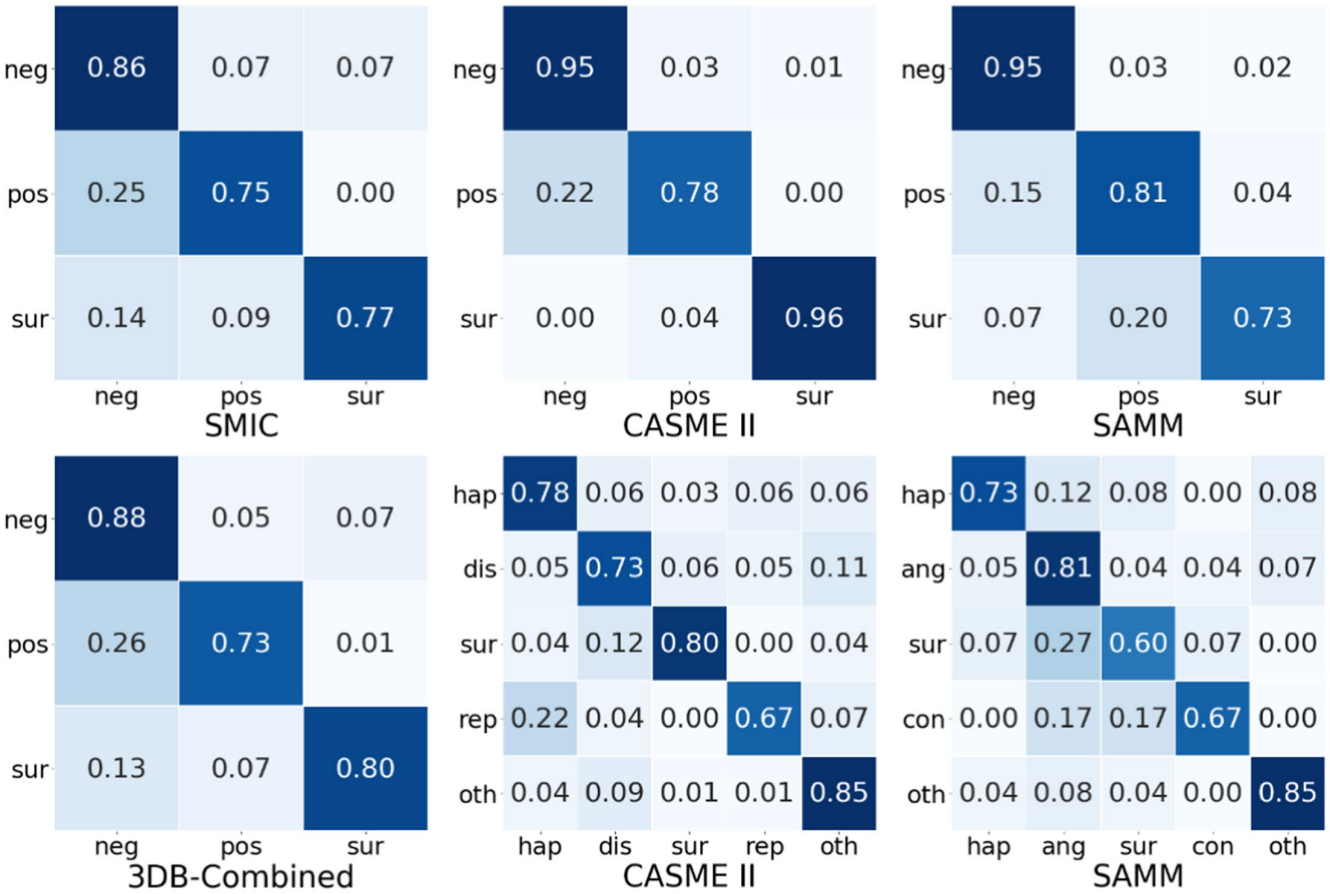
The confusion matrices on of MFVAN model on micro-expression datasets.

This section further uses the confusion matrix of the MFVAN model on the SMIC, CASME II, SAMM, and 3DB-Combined datasets to visually analyze the recognition performance of different types of micro-expressions. The experimental results are shown in Figure 5. In general, whether it is a single data set or a combined data set, the performance of the negative type is higher than the emotional performance of the positive type. The experimental results mainly include two reasons. First, in the process of constructing the micro-expression dataset. The negative emotion category of the subject is more likely to be stimulated, making the samples of negative emotions in the data set higher than the samples of positive emotions; on the other hand, the reason is that the MFVAN model tends to be more inclined to negative emotional types, this is exactly one of the problems that need to be solved in the follow-up.

**Figure 6:**
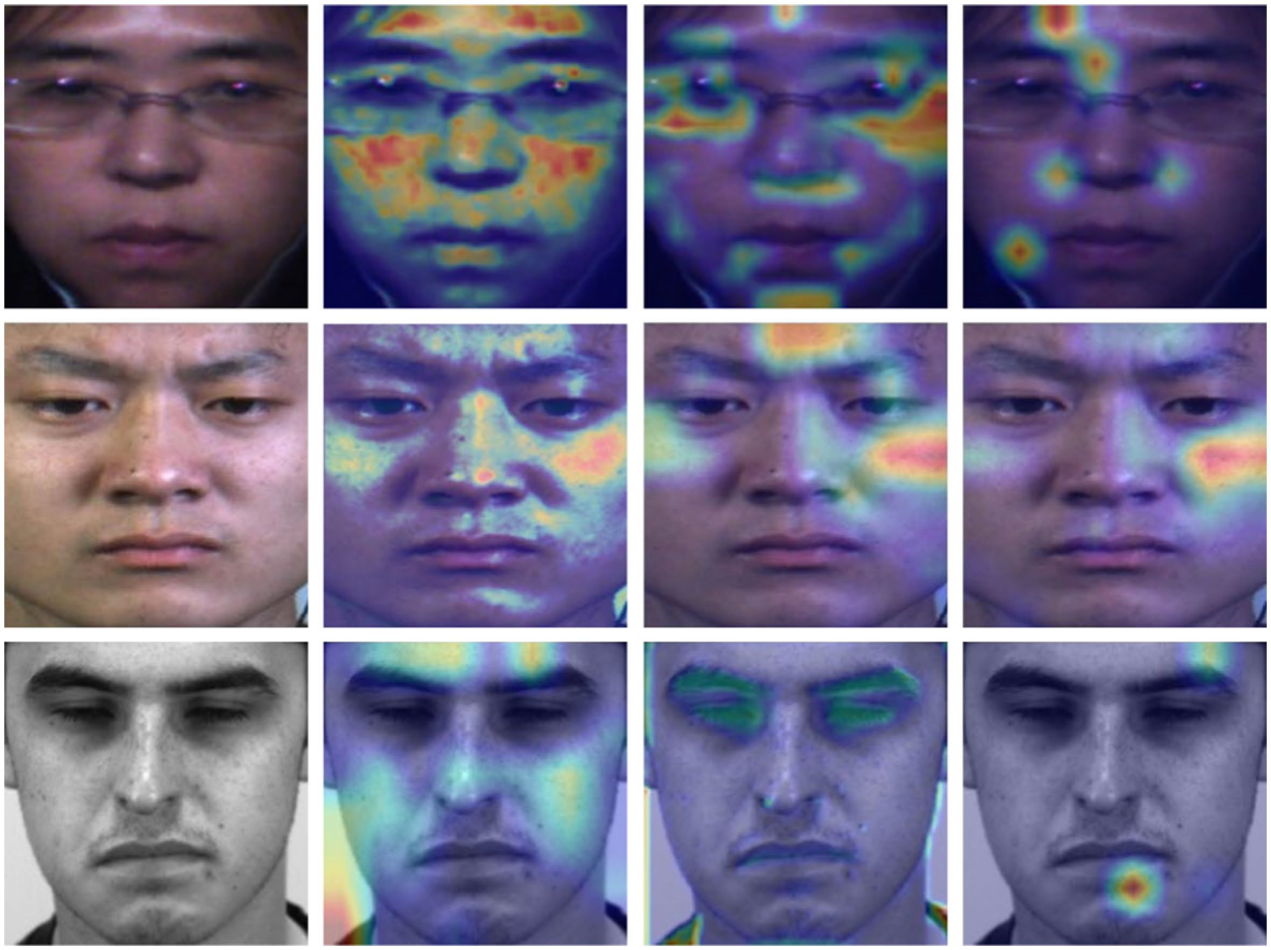
The Grad-weighted Class Activation Mapping of the multi-scale features on the SMIC, CASME II, and SAMM.

We visualized analysis of the effect of multi-scale features on the micro-expression recognition through the Grad-weighted Class Activation Mapping (Grad-CAM) (Selvaraju et al., 2017). The experimental results are shown in Figure 6. The first column is the original image. The second column is the category activation map corresponding to the local features at the scale of the original image. The third and fourth columns are the category activation maps corresponding to the first and second convolutional feature map scales. In terms of the performance on the multi-scale local feature of the class activation maps corresponding to single-scale features, the local attention weights focused are more dispersed in the original image scale. And the local features at small scales are relatively more concentrated. Therefore, it is necessary to fuse local features from multiple scales to recognize micro-expressions.

## 5. Conclusion

In this paper, we propose a multi-scale fusion visual attention network model that fuses the local attention weights of the multiple-scale feature maps of the removing identity attributes network for micro-expression recognition. For the problem of the small micro-expression dataset, a combination of unsupervised and transfer learning is used to reduce the influence of identity attributes by learning the mapping relationship from apex to onset frame in micro-expression video sequences. Then, the local detail features are extracted by focusing on multi-scale local attention weights. Finally, micro-expressions are classified by fusing global features with local features with high weights. In general, we reveal the impact of individual attributes on the localization of local ROIs. The experimental results show that a multi-scale fusion visual attention network contributes to micro-expression recognition.

The research work related to micro-expression analysis in this paper mainly discusses the micro-expression recognition problem, but often there is still how to locate the occurrence of micro-expressions in the real environment. In a real environment, the occurrence of micro-expressions is often to conceal true emotions, so micro-expressions are often accompanied by the occurrence of macro-expressions. How to locate the location of micro-expressions in a complex environment and emotional changes is also important to research in future work.

## Data availability statement

The original contributions presented in the study are included in the article/supplementary material, further inquiries can be directed to the corresponding author.

## Author contributions

HY: data curation, software, validation, investigation, writing—original draft preparation, and visualization. HP: methodology, software, validation, and visualization. LX: conceptualization, formal analysis, investigation, resources, and funding acquisition. ZW: resources and visualization. All authors contributed to the article and approved the submitted version.

## Funding

This work was supported in part by the National Key R&D Program of China under Grant 2018YFC2001700 and in part by the Beijing Natural Science Foundation under Grant L192005.

## Conflict of interest

The authors declare that the research was conducted in the absence of any commercial or financial relationships that could be construed as a potential conflict of interest.

## Publisher’s note

All claims expressed in this article are solely those of the authors and do not necessarily represent those of their affiliated organizations, or those of the publisher, the editors and the reviewers. Any product that may be evaluated in this article, or claim that may be made by its manufacturer, is not guaranteed or endorsed by the publisher.
